# The neurobiology of social stress resulting from Racism: Implications for pain disparities among racialized minorities

**DOI:** 10.1016/j.ynpai.2022.100101

**Published:** 2022-08-20

**Authors:** Joanna M. Hobson, Myles D. Moody, Robert E. Sorge, Burel R. Goodin

**Affiliations:** a Department of Psychology, University of Alabama at Birmingham, Birmingham, Alabama, U.S.A; b Department of Sociology, University of Alabama at Birmingham, Birmingham, Alabama, U.S.A; c Center for Addiction & Pain Prevention & Intervention (CAPPI), University of Alabama at Birmingham, Birmingham, AL, U.S.A

**Keywords:** Racism, Pain, Brain networks, Disparities, Social threat

## Abstract

Extant literature posits that humans experience two types of threat: physical threat and social threat. While describing pain as “physical” or “social” can be helpful for understanding pain origins (i.e., broken bone versus lost relationship), this dichotomy is largely artificial and not particularly helpful for understanding how the human brain experiences pain. One real world example of social exclusion and rejection that is threatening and likely to bring about significant stress is racism. Racism is a system of beliefs, practices, and policies that operates to disadvantage racialized minorities while providing advantage to those with historical power, particularly White people in the United States and most other Western nations. The objective of this Mini-Review is to present evidence in support of the argument that racism promotes physical pain in racialized minorities, which in turn promotes chronic pain disparities. First, we provide a theoretical framework describing how racism is a potent stressor that affects the health and well-being of racialized minorities. We will then address the neurobiological underpinnings linking racism to social threat, as well as that linking social threats and physical pain. Finally, we will discuss how the perception of social threat brought about by racism may undermine pain management efforts.

## Highlights

• There is a bi-directional relationship between experiences of social and physical pain, and this relationship may be explained by shared neural circuitry (i.e., anterior insula, dorsal anterior cingulate cortex, amygdala, etc).
• Racism is a social construct that can be perceived as threat, thus over activating stress-regulatory systems and inducing or exacerbating physical pain.
• It is common for minoritized individuals to experience racism in their daily lives, as well as in experimental and healthcare settings.
• Social stress due to racism may be a potential explanation for racial disparities in pain conditions.

## 1 Introduction

The International Association for the Study of Pain (IASP) recently updated the definition of pain to reflect “an unpleasant sensory and emotional experience that is associated with, or resembling that associated with, actual or potential tissue damage (Raja et al., 2020 Sep).” Despite the best efforts of this updated pain definition, many people, including healthcare providers, researchers, and laypersons, continue to view the emotional experience as merely a reaction to pain resulting from actual or potential tissue damage (i.e., physical pain). Indeed, negative emotional responses often worsen as pain becomes chronic; however, current neuroscientific evidence suggests that the relationship between the experience of pain and negative emotions is bidirectional (Gilam et al., 2020 Jul 8). Importantly, negative emotions can cause a human being to experience pain irrespective of the presence of actual or potential tissue damage (Garland, 2012 Sep, Lumley et al., 2011 Sep). The experience of pain and negative emotions reinforce each other in humans and involve activation of the same brain structures (Etkin et al., 2011 Feb, Price, 2000 Jun 9).

In everyday life, humans can experience two types of stressors: physical pain and social threats (Eisenberger, 13). As the updated IASP definition suggests, physical pain is associated with actual or potential tissue damage. Social threat, however, is conceptualized as the painful experience of actual or potential interpersonal rejection or intentional abuse. The hallmark of social threat is the negative emotions brought about by ostracism and either the inability to attain desired relationships or the loss of desired relationships (MacDonald and Leary, 2005 Mar). The pain of a broken heart can be just as averse and emotionally distressing as the pain of a broken arm. While describing pain as “physical” or “social” can be helpful for understanding pain origins (i.e., lost relationship versus broken bone), this dichotomy is mostly artificial and not particularly helpful for understanding how the human brain experiences pain. This is partially explained by the similarities in brain circuitry responsible for mentalizing, the affective component of pain, and the mirror network (e.g., insula, dorsal anterior cingulate cortex, medial and ventral prefrontal cortex) (Eisenberger, 13, Zhang et al., 2019 Dec 1). Along this line, many modern functional neuroimaging studies have now shown that social threats affect the perception of physical pain, and these two types of stressors look quite similar in brain activation images (Masten et al., 2011 May 1, Eisenberger et al., 2007, Eisenberger et al., 2006 Dec 15, Losin et al., 2020 May).

One real world example of social threat due to exclusion and rejection is racism. Racism is a system of beliefs, practices, and policies that operates to disadvantage racialized minorities while providing advantage to those with historical power, non-Hispanic White (NHW) people in the United States and most other Western nations (Goosby et al., 2018 Jul, Harrell, 2000 Feb). Hallmark aspects of racism include racial prejudices (opinions, beliefs, attitudes, or assumptions about racialized groups based on stereotypes and often without firsthand experience) and racial discrimination (differential treatment of a person or group of people based on prejudices of their presumed racial group) (Haeny et al., 2021 Sep). Therefore, we posit that racism promotes social stress and is perceived as a social threat via the social rejection and exclusion of racialized minorities, and may explain why racialized minorities tend to experience greater (physical) pain disparities (Strand et al., 2021 Jun 1, Janevic et al., 2017 Dec). While the contribution of racism to the perpetuation of pain disparities is a topic of growing interest, to date it has received little research attention. However, the broader health literature attests to the deleterious effects of racism on physical and mental health (Jackson et al., 1996, Williams and Williams-Morris, 2000 Nov, Williams, 2018 Dec). It therefore stands to reason that racism is a potent contributor to physical pain disparities among racialized minorities through the elicitation of social stress brought on by racism.

## 2 Minority stress theory

The minority stress theory is a conceptual framework that explains how minority and minoritized groups are exposed to routine inequities and disadvantages (e.g., stigma and discrimination, financial hardships, limited access to resources), and these exposures may lead to an accelerated decline in their physical and mental health (Forde et al., 2019 May, Geronimus, 1992, Geronimus et al., 2006 May, Meyer, 2003 Sep, Velez et al., 2013 Jul). Underlying assumptions of minority stress are that it is (a) unique (additive to general stressors that all people experience, leaving stigmatized groups with the task of using extra effort to adapt to those stressors, (b) chronic (related to social and cultural structures) and (c) socially-based (stemming from social processes, institutions and structures beyond the individual rather than general events/conditions that induce interpersonal stress or other nonsocial characteristics of that individual/group (i.e. biological or genetic factors)) (Meyer, 2003 Sep). Originally, the minority stress theory was centered on stress, coping, and the mental health outcomes of sexual minorities (i.e. lesbians, gay males and bisexuals). It was conceptualized that stress is experienced in minorities’ natural environments, and can be influenced by factors like socioeconomic status and minority/minoritized status (e.g. sexual orientation, race/ethnicity, gender). Taken together, these factors would influence an individual’s exposure to stressors and coping resources (Díaz et al., 2001 Jun). Individual environments can influence exposure to stressors, including general stressors (i.e. loss of a job or a loved one) and minority stressors (i.e. discrimination, antigay or race-related violence). Minority stressors, including stigma and discrimination, may contribute to increased vigilance and anticipation of social rejection (Hicken et al., 2013 Jun 1, Hicken et al., 2014 Jan, Lee and Hicken, 2016, Powell et al., 2016 Jun, Sawyer et al., 2012 May).

Specifically related to racialized minorities and people of color, the minority stress theory is supported by the “weathering hypothesis” (James et al., 1992). This hypothesis posits that non-Hispanic Black (NHB) and Brown individuals experience earlier declines in health than NHW individuals due to the constant exposure to social and economic inequities. The persistent use of high-effort coping (e.g. John Henryism) to combat acute and chronic stressors have disproportionate effects on the health of NHB individuals, such that a middle-aged NHB individual has been shown to exhibit the health characteristics of a NHW individual who is significantly older (Geronimus et al., 1996 Nov 21). This accelerated decline in health among NHB individuals accumulates over time, creating substantial racial disparities in health by middle adulthood (Forde et al., 2019 May, Schoendorf et al., 1992 Jun 4, Anderson et al., 2009 Dec 1).

### Allostatic load

Inequalities in resources and unequal life experiences contribute to health disparities amongst racial minorities in the United States (Meloni, 2017). Currently, there is no consensus on the debate of race as a biological construct versus a social construct (Meyer, 1995). Although prior literature has identified race as a social construct associated with skin color, evidence from the minority stress theory has posited that the lived experiences of NHBs tend to be more exhausting than NHWs and has been reflected biologically as allostatic load (James et al., 1992, McEwen and Wingfield, 2003 Jan 1). Allostasis is the relationship between stress and physiological functioning, and involves the body’s response to chronic stress via the regulation of homeostasis (McEWEN, 1998). Although the human body is generally able to adapt to stressful life events, chronic exposure to stress over time can have significant “wear and tear” on the body, and could eventually lead to the dysregulation of multiple physiological systems (e.g. cardiovascular, metabolic, inflammatory, neuroendocrine), functional decline, development and/or exacerbation of diseases and, ultimately, death (James et al., 1992, Schoendorf et al., 1992 Jun 4, McLean et al., 2020 Feb, Mcewen, 1998). When stress-related systems are routinely over-worked and not allowed adequate time for recovery, patterns of dysregulation in these systems become evident, indicating high allostatic load. Over time, the subtle dysregulation of multiple systems may contribute to poor health outcomes. This is especially important for NHBs, as prior literature has shown that they display higher allostatic load than NHWs, and this disparity becomes more robust with age (Geronimus, 1992).

Measurements of allostatic load can be broken down into two categories: primary mediators and secondary outcomes. Primary mediators are the substances that are released from the body in response to stress, such as norepinephrine, epinephrine, cortisol, and dehydroepiandrosterone sulfate (DHEA-S) (Rodriquez et al., 2019 Mar). Consequences of the primary mediators include elevated levels of systolic and/or diastolic blood pressure, cholesterol, glycated hemoglobin, and waist-to-hip ratios (Geronimus et al., 2006 May). Taken together, these markers have been used to calculate a measure of allostatic load, representing the physiological burden of chronic stress. Researchers have also elucidated additional markers of allostatic load including dopamine, insulin-like growth factor-1, fasting glucose, triglycerides, apolipoprotein A1, body mass index (BMI), C-reactive protein and waist circumference (McEwen, 2003 Oct, Totsch et al., 2017). Several physiological systems and pathways are represented in the overall measure of allostatic load, including the cardiovascular (e.g. systolic and diastolic blood pressure, lipid levels), metabolic (e.g. glycated hemoglobin, waist-hip ratio, body mass index), inflammatory (e.g. CRP, IL-6, fibrinogen, insulin-like growth factor 1) and neuroendocrine (cortisol, epinephrine, norepinephrine and DHEA-S) systems. Given the known associations between these biomarkers and pain (Timmers et al., 2019 Dec, Úbeda-D’Ocasar et al., 2020, Clark et al., 2013) it is feasible to believe that chronic stressors over-activate these regulatory systems, resulting in a dysfunction in circulatory markers and hypersensitivity to later pain.

### 2.1 Defining racism

Racism is a system of dominance, power and privilege that is deeply rooted in the historical oppression of racialized minority groups and is manifested by maintaining power, esteem, status and unequal access to societal resources (Harrell, 2000 Feb). It has been suggested there are 3 forms of racism: individual, institutional, and cultural that converge and manifest into interpersonal, collective, cultural-symbolic and sociopolitical contexts. Interpersonal racism involves direct and indirect experiences of prejudice and discrimination (e.g., interactions with others, observing their actions, nonverbal behavior and verbal statements). Collective racism involves racial inequities in educational achievement, (un)employment rates, incidence and prevalence of disease, and treatment in the criminal justice system. Cultural-symbolic racism involves the mockery of non-dominant racial groups through media and entertainment, art and literature, as well as research and scientific inquiry. One example of cultural-symbolic racism is the belief that there are biological differences between Blacks and Whites. Lastly, sociopolitical racism involves political decisions and legislative processes regarding race that unduly benefit the racialized majority.

Racism affects the well-being of racialized minority groups via stress, as well as its influence on mediators of stress such as coping and support resources (Essed, 1991). Individuals who experience racism often have their experiences invalidated, and this invalidation involves others challenging the reality or significance of their experience (Mental and Health, 2016). Thus, there can be a compounding effect of racism including the stress related to the actual experience, as well as the stress resulting from experiential invalidation from others (Wheaton, 1994). This process of having an individual’s experience and perceptions challenged, ruminating over the experience, attempting to explain it to others and entertaining other explanations (e.g. gaslighting) can induce stress beyond an individual's adaptive ability (Harrell, 2000 Feb, Haeny et al., 2021 Sep, Wheaton, 1994).

There are 6 types of racism-related stress that fall under the general stress processes of episodic stress (direct and indirect racism), daily hassles (racial micro-aggressions) and chronic strain (chronic-contextual, collective, and transgenerational transmission) (Williams et al., 2010 Feb). Examples of direct racism-related life events are being rejected for housing/a loan, receiving poor service in a store/restaurant, or being harassed by the police (Nadal, 2011). These incidents are less likely to occur as frequently as daily hassles, but still contribute to stress for racialized minorities. Vicarious experiences of racism involve the indirect effect of racism through watching close family members/friends experience discrimination, as well as watching it on various forms of media (Harrell, 2000 Feb, Mental and Health, 2016). These experiences can create second-hand anxiety, vigilance, anger and sadness among many other emotional reactions. Daily hassles occur more frequently, and involve microaggressions such as being ignored/overlooked while waiting in line, being mistaken for someone that serves others, and being followed or watched in public establishments (Sue et al., 2007, Brondolo et al., 2011 May). These experiences can be extremely humiliating and disrespectful and are often overlooked by racial minorities as a means of coping, which over time, can lead to weakening of stress-regulatory systems as well as vulnerability to future stressors (Harrell, 2000 Feb).

In addition to the stress resulting from day-to-day living (e.g., traffic, workload, family arguments), racism-related stress adds an additional burden to racialized minorities that further impacts their health. Racism has the ability to affect health via physical, psychological, social, functional, and spiritual domains. Specifically, racism-related stress has been related to hypertension (Williams and Neighbors, 2001, Anderson, 1989), cardiovascular issues (Paradies et al., 2015 Sep 23), depression (Willis et al., 2021 Aug 1), psychological distress (Jackson et al., 1996, Kelly et al., 2020 Apr), and health behaviors such as eating disorders (Glass et al., 2020 Nov), substance abuse (Jones et al., 2019 Jun, Desalu et al., 2019 Jun, Frazer et al., 2018 Feb) and violence (Sutton et al., 2020 Feb, Johnson et al., 2021). Additionally, racism-related stress has effects on social (ability to trust and interact with others) (Hicken et al., 2013 Jun 1, Odoms-Young, 2018), functional (academic and financial achievement) (Grace and Nelson, 2019 Oct 2, Butler-Barnes et al., 2018 Oct) and spiritual (faith in higher powers) well-being (Drolet and Lucas, 2020 Dec, Bailey et al., 2020). Mediators of racism-related stress on health can be internal (coping behaviors, appraisal, self-esteem, affective reactions) or external (social support), and can alternatively influence the health outcomes of racialized minority groups (Harrell, 2000 Feb).

### 2.2 Racism and racial discrimination as social threat

Social stressors such as racism are most impactful when experienced firsthand and perceived as a social threat. (Goosby et al., 2018 Jul). The United States has a long history of pervasive racism in society that continues to impact how people perceive race and racial identity today (Hoffman et al., 2016 Apr 19). Many Americans develop racial prejudices, which are beliefs about a person based on stereotypes of their presumed racial group/identity (Harrell, 2000 Feb, Haeny et al., 2021 Sep). For example, studies have shown that medical professionals have false beliefs about biological differences between NHB individuals and NHW individuals, and these beliefs influence treatment for pain conditions (Sellers et al., 2003 Sep). Racial prejudices can contribute to racial discrimination, which refers to the unfair and/or mistreatment of a person based upon their presumed racial group/identity (Berger and Sarnyai, 2015 Jan 2). Regardless of whether racial discrimination is perpetrated covertly or overtly as a microaggression or direct threats or acts of rejection, respectively, it represents a potent social threat given the possibility for physical assault and verbal abuse, ostracism and exclusion (Goosby et al., 2018 Jul, Harrell, 2000 Feb). There are various physiological processes that are activated once racism and racial discrimination are perceived and, over time, the over-activation of these physiological systems can have adverse consequences for brain structure and function (Goosby et al., 2018 Jul, Tull et al., 2005 Feb). Specifically, racial discrimination is perceived as a form of social threat that simultaneously activates the hypothalamic–pituitaryadrenal (HPA) axis (Tull et al., 2005 Feb, Bell et al., 2019 Jun), the autonomic nervous system (ANS) (Cheadle et al., 2020 Dec 8, Peterson et al., 2020 Sep 2) and initiates the release of stress-related hormones (e.g., cortisol) (Bell et al., 2019 Jun, Lee et al., 2018 Apr 1, Kapuku et al., 2002 Nov 1, Goldstein and McEwen, 2002 Jan 1). Specifically, chronic cortisol elevation over time contributes to allostatic load (described in greater detail below) (Rodriquez et al., 2019 Mar, Totsch et al., 2017, Tull et al., 2005 Feb, McEwen and Gianaros, 2010 Feb, Berger and Sarnyai, 2015 Jan) and, when this elevation occurs simultaneously with chronic ANS responses, can contribute to changes in various brain networks that impact physical and mental health (Goosby et al., 2018 Jul, Corbetta et al., 2008 May 8). One brain network that may be especially susceptible to social threat and rejection of racial discrimination is the salience network. The salience network is a collection of regions of the brain that select which stimuli are deserving of attention. The network has key nodes in the insular cortex, is critical for detecting behaviorally-relevant stimuli and for coordinating the brain’s neural resources in response to these stimuli (Seeley et al., 2007 Feb 28, Eisenberger and Lieberman, 2004 Jul 1, Dedovic et al., 2009 Oct). Components of the salience network, particularly the dorsal anterior cingulate cortex (dACC) and dorsomedial pre-frontal cortex (DMPFC), receive cortisol inputs that can contribute to glucocorticoid-driven structural changes within the salience network, as well as altered connectivity due to the elevated influx of cortisol (Schmitt et al., 2014 Jul). In turn, these social threat-related structural and connectivity changes in the salience network - in addition to HPA-axis dysregulation - can lead to hypervigilance, which can result in increased vulnerability to subsequent social threats (Tull et al., 2005 Feb). These processes contribute to a variety of deleterious physical and mental health consequences such as anxiety and depression disorders (Willis et al., 2021 Aug 1, Misiak et al., 2014 Sep), schizophrenia (Edwards, 2008 Jun 25), chronic pain (Burgess et al., 2009 Nov 1, Wagner et al., 2015 Aug), high blood pressure, cardiovascular disease, diabetes and obesity among others (Goosby et al., 2018 Jul, Herman and Panksepp, 1978 Aug 1).

## 3 Social safety theory & pain overlap Theory: Why does racism hurt?

We contend that racism-related stress is derived (in large part) from the social stress, rejection, and exclusion of racialized minorities who experience racial discrimination and, in turn, culminates in the experience of social threat. The overlap between social threat and physical pain is based upon the idea that experiences of social rejection or exclusion are perceived as “painful” because: 1) these experiences are processed by many of the same neural regions that process physical pain via threat-detection mechanisms (pain overlap theory), and 2) over time, threats to social connections are perceived as threats to survival (social safety theory) (MacDonald and Leary, 2005 Mar, Zhang et al., 2019 Dec 1). From an evolutionary perspective, natural selection and the neural underpinnings of human social attachment are shared with many of the neural substrates responsible for physical pain processing. For this reason a pain signal can be initiated when social connections are threatened (Panksepp et al., 1978, Panksepp, 2004). For prehistoric humans, social connection was important for the survival of the species, particularly those who lacked the capacity to feed or defend themselves when faced with predators. Therefore, humans had to develop effective social connections within their communities in order to survive (Gilbert, 2016, Whiten et al., 1989, Thornhill and Thornhill, 1989). Being separated from others, ostracized, or rejected decreased the chances of survival and, as a result, humans became highly motivated to avoid situations that threatened social ties or led to rejection (Kling et al., 1970, Tremblay and Sullivan, 2010 Feb 1). In essence, the perception of social threat following experiences of social rejection or exclusion may serve as a protective/adaptive response that protects against further social rejection. This is similar to how physical pain serves as a signal that elicits adaptive responses that promote tissue health as well as avoidance of further injury and other threats to safety (MacDonald and Leary, 2005 Mar, Kling et al., 1970). Below we provide evidence from the extant pharmacological, behavioral, and social neuroscience literature demonstrating the neural underpinnings of physical pain and social threat, as well as potential implications for pain disparities and pain management.

### 3.1 Social safety Theory: Cumulative effects of biopsychosocial risk factors on human health and behavior

One of the most important and impactful theories that underlies the potential mechanism whereby racism contributes to pain disparities is the social safety theory. The social safety theory posits that the relationship between the human brain and immune system have one parallel goal: to keep the body biologically and physically safe. In efforts of doing this, humans remain internally and externally vigilant about maintaining friendly bonds and reducing exposure to threats that increase risk for physical injury and infection over time. Thus, the human brain continuously monitors the social environment, interprets social cues and behaviors, and determines whether the surroundings are safe. To do this, the brain uses 4 major social networks: the amygdala network, the mentalizing network, the empathy network and the mirror network. Additionally, when the brain perceives a social threat, there is multilevel activation of the sympathetic nervous system (SNS), the HPA axis, the vagus nerve and the meningeal lymphatic vessels. These pathways allow the bi-directional communication between the brain and immune system, and contribute to short-term protective factors, and long-term disease risk, illness and mortality. Specifically, the short-term benefits of activating these pathways are heightened awareness of threat and pain sensitivity, avoidance of physical harm, enhanced wound healing, improved physical recovery and greater likelihood of survival (Harrell, 2000 Feb). In turn, the long-term costs of over-activating these multilevel systems can be anxiety, hypersensitivity to pain, depression, greater inflammatory disease risk, vulnerability to infection, accelerated aging and early death. Compounding these factors are also other biopsychosocial factors such as birth cohort, adverse childhood experiences, sleep, genetics, air pollution, diet, and self-harming behaviors such as substance usage.

### 3.2 How does social threat contribute to physical pain & vice versa?

One of the most intriguing insights from the physical-social threat overlap is the development of two hypotheses: The individual differences hypothesis and the manipulation hypothesis (Eisenberger, 13). The **individual differences hypothesis** states that individuals who are more sensitive to physical pain should also be more sensitive to social threat due to the similar underlying processes that are shared between both experiences. Evidence for this hypothesis has been shown in multiple studies. For example, one study found that individuals who showed greater sensitivity to heat pain expressed greater social distress after being excluded from a game of cyber-ball (Eisenberger et al., 2006 Dec 15). Other studies have found associations between pain sensitivity and attachment anxiety among adolescents (Ehnvall et al., 2009), as well as rejection sensitivity and pain symptoms among depressed individuals (Chalmers et al., 1995 Dec). Thus, individuals who are more sensitive to one type of threat (social versus physical) may also be more sensitive to the other. The **manipulation hypothesis** states that factors that either increase or decrease physical pain should have similar effects on social threat. This hypothesis has been tested in studies examining associations between physical pain and social support (Kennell et al., 1991 May 1, King et al., 1993, Kulik and Mahler, 1989, López-Martínez et al., 2008 Apr) as well as inflammation and social disconnection. For example, one study of individuals with chronic pain found that those who reported higher levels of social support experience less pain-related distress and less pain (Penn et al., 2019 Apr). Another study of HIV + individuals found that social support buffered the relationship between perceived injustice and pain interference, such that greater social support lessened the effects of injustice on the perception of pain (Crockett and Turan, 2018 Dec). Similar associations between social support and pain intensity have been shown in other studies of HIV + individuals (Jensen et al., 2011 Jan). Specifically, social support has been shown to promote healthy coping and mitigates the impact of stressful events on health (Eisenberger et al., 2003 Oct 10). Thus, the manipulation hypothesis serves as a framework to support the notion that factors that often influence one source of pain may have similar effects on the other.

### 3.3 Racism and racial discrimination effects on social and physical threat brain networks

Many of the same brain regions and networks vital for processing social and physical threats show similar activity in response to racism and racial discrimination. Studies using functional magnetic resonance imaging (fMRI) have shown increased activity in the ACC, the AI, and the prefrontal cortex (PFC) following race-related discriminatory experiences (Masten et al., 2011 May 1). Studies have also provided evidence for a regulatory role of the PFC (ventrolateral and dorsolateral) in the negative affective responses to socially painful threats, as implicated by correlated reductions in dACC and AI activity (Lieberman et al., 2004 May 1, Wager et al., 2004 Feb 20, Petrovic and Ingvar, 2002 Jan, Clark et al., 2018 Apr 1). The amygdala has also been shown to play a role in experiences of racial discrimination and social exclusion/rejection. Specifically, one study found that greater social discrimination exposure (due to race/ethnicity, gender, sexuality) was associated with higher levels of spontaneous amygdala activity, as well as stronger functional connections between the amygdala and several regions (AI, ACC, putamen, caudate, thalamus and medial frontal gyrus) independent of other demographic and psychological factors (Krill and Platek, 2009). Other studies have also shown increased amygdala reactivity in individuals who are targets of social exclusion and rejection (Eisenberger et al., 2007, Tawakol et al., 2017 Feb 25). Given the amygdala’s role in the experience of psychosocial stress, physiological arousal, hypervigilance, and threat detection (Muscatell et al., 2015 Jan, Hermans et al., 2014 Jun 1, Machulda et al., 2011 Sep 1, Green et al., 2003 Sep 1), it is not surprising that this brain region is similarly active in response to discrimination and related social rejection/exclusion, as well as the affective-motivational aspect of physical pain processing. Taken together, many of the brain regions and networks that process fear and negative emotions associated with physical pain (amygdala, dACC, AI, dmPFC) are the same regions and networks that process fear and negative emotions associated with racism-related social exclusion and rejection. The amygdala, dACC, AI, and dmPFC are responsible for monitoring and responding to the social environment, including threats to inclusion, and interact with key brain regions that mediate stress response systems. The interactions between these brain networks allow for the processing of the social environment, influence future social interactions, and enable the anticipation of potential threats involved in future encounters (Dedovic et al., 2009 Oct).

## 4 Implications for racial disparities in pain

Racial disparities in the prevalence and treatment of chronic pain have long been documented (Edwards et al., 2001 Apr). NHB individuals experience greater pain sensitivity and intensity, as well as greater pain-related physical disability than their NHW counterparts, yet their pain is still underestimated and undertreated in healthcare settings (Riley et al., 2002 Nov 1, Green et al., 2003 Mar 1). Beyond the physical limitations of chronic pain, NHB individuals have also reported greater symptoms of posttraumatic stress disorder, irritability, depressive symptoms, hypervigilance and disability related to their chronic pain condition than NHW individuals (Green et al., 2003 May 1, Campbell et al., 2005 Jan 1, Chapman and Jones, 1944 Jan 1). Thus, the burden of chronic pain disproportionately affects NHB individuals in comparison to their NHW counterparts. These findings have been replicated in experimental settings as well, such that NHB individuals have demonstrated lower heat pain threshold and tolerance (Woodrow et al., 1972 Nov), lower pressure-pain (Walsh et al., 1989 Feb 1), and cold-pain tolerance (Mathur et al., 2014 Apr 1), as well as greater pain-induced distress in comparison to NHW individuals (Chapman and Jones, 1944 Jan 1). Thus, there are racial differences in pain sensitivity. These differences are thought to be generated by a variety of factors (e.g., patient-level factors, provider-level factors, and healthcare system related factors) that ultimately complicate pain assessment, management and prevention for NHB individuals.

### 4.1 Racial discrimination in everyday life influences pain

NHB individuals experience more racial discrimination in the form of microaggressions, aggressive policing, residential segregation, poor access to resources (e.g., healthcare and education) and racial profiling than their NHW counterparts (Goldstein and McEwen, 2002 Jan 1, McEwen and Gianaros, 2010 Feb, Berger and Sarnyai, 2015 Jan, Corbetta et al., 2008 May 8). As such, racial discrimination and related stress has been shown to be associated with chronic pain severity and experimental pain sensitivity (Losin et al., 2020 May). For example, a study of 393 NHB male veterans found that those who reported greater experiences of daily discrimination also reported greater bodily pain (Wagner et al., 2015 Aug). These findings have been replicated amongst individuals with chronic low back pain (Riley et al., 2002 Nov 1) and painful sickle cell disease (Goodin et al., 2013 Nov). Additional studies incorporating quantitative sensory testing for the assessment of pain sensitivity found that NHBs reported greater perceived racial discrimination and greater mistrust of medical researchers compared to NHWs. Additionally, greater experiences of racial discrimination were associated with decreased heat pain tolerance in NHB individuals but not NHW individuals with symptomatic knee osteoarthritis (Bräscher et al., 2016 May 4). In a recent laboratory study of 97 chronic pain-free adults, it was found that NHB individuals demonstrated significantly greater experimental pain sensitivity to a noxious thermal stimulus compared to NHW individuals (Losin et al., 2020 May). Interestingly, this racial difference was mediated by experiences of racial discrimination, which correlated with increased activity in the medial PFC, ventromedial PFC, nucleus accumbens (NAc), and ventromedial PFC-NAc pathway in response to thermal noxious stimulation. Additionally, increased activity in these brain regions was associated with greater pain intensity ratings, greater experiences of racial discrimination, and experimenter mistrust (Bräscher et al., 2016 May 4). These findings bring about new insights into the possible relationships linking racial discrimination to pain via racism-related stress. This is because increased activity in the medial PFC/ventromedial PFC-NAc pathway has also been associated with chronic stress, and increased connectivity with insular regions when pain is uncontrollable, further contributing to the distress of having chronic pain (Dias-Ferreira et al., 2009 Jul 31, Race, 2001). It is reasonable to believe that greater activity in stress-related neural pathways may be related to racial discrimination, and this brain activity may result in altered appraisals of painful stimuli as more threatening and intense, thereby promoting social threat and further contributing to disparities in pain reports amongst NHB individuals.

### 4.2 Racial bias in pain treatment exacerbates pain conditions

Historically, scientists and healthcare workers have underestimated and undertreated the pain of NHB individuals in comparison to other racial groups (Cintron and Morrison, 2006 Dec 1, Shavers et al., 2010, Nelson, 2002 Aug, Todd et al., 2000 Jan 1, Staton et al., 2007 May, Goyal et al., 2015 Nov 1). It has been shown that medical trainees (i.e., medical students and residents) have false beliefs and preconceived notions related to biological differences between NHB and NHW individuals that differentially influence pain treatment efforts. In this study by Hoffman and colleagues it was revealed that laypersons and medical trainees alike believed that NHB individuals have “thicker skin”, feel less pain, have greater pain tolerance and less sensitive nerve endings than NHW individuals (Green et al., 2007 Sep 1). In turn, these false beliefs resulted in differential pain treatment between NHB and NHW individuals, thereby perpetuating pain disparities. These results come as no surprise as further evidence has shown that NHB patients are less likely to be given pain medications – especially opioid analgesics - than NHW individuals and, if given medications, they receive lower quantities. To illustrate, one study found that compared to NHW patients, NHB patients were significantly less likely to receive analgesic medications for extremity fractures in the emergency room despite having similar pain reports (Goyal et al., 2015 Nov 1). Another study found that physicians were more likely to underestimate the pain of NHB patients in comparison to NHW patients (Guillory, 1968). These disparities in the assessment and treatment of pain have also been replicated amongst NHB children (Medical, 2006). Thus, regardless of their age, NHB individuals are more likely to be undertreated for their pain and have their pain conditions underestimated. Although previous research has implied that these disparities in pain assessment and treatment are due to concerns about non-compliance and access to healthcare and not racist beliefs, there is evidence that implicit pro-NHW attitudes have influenced the likelihood of ineffective pain treatment amongst NHB individuals (Diniz et al., 2020 Feb). Additionally, the belief that race is a biological construct is deeply rooted in the injustices and implicit biases brought about by slave owners (to justify slavery), and later incorporated by some healthcare providers and researchers to justify the inhumane treatment of NHB individuals in medical research (Devan et al., 2021 Oct 8, Zajacova et al., 2022). Discrimination is deeply rooted in power dynamics and results in the withholding of resources. As such, it is reasonable to believe that discriminatory attitudes among healthcare providers, whether conscious or subconscious, may contribute to the undertreatment of pain in racialized minorities, which in turn perpetuates pain disparities. This is not only an issue for NHBs, but for other racial and ethnic minority groups as well (146–148).

## 5 Summary

In summary, racism is a threatening social phenomenon that promotes social rejection, ostracism, and exclusion through racial prejudices (beliefs) and racial discrimination (behaviors). We argue that racism is likely a potent contributor to social threat, which is known to exacerbate physical pain conditions and contribute to adverse physical and mental health outcomes. Brain imaging studies using both laboratory animal models and humans have found similar neural underpinnings of social threat and physical pain. Further, recent research has shown that experiences of racial discrimination activate many of the same brain regions that are implicated in the affective-motivational processing (i.e., unpleasantness) of physical pain, and more recently, also the sensory-discriminative processing (i.e., intensity) of physical pain. Experiences of racial discrimination have also been associated with increased clinical pain severity, increased experimental pain sensitivity, and increased pain catastrophizing amongst NHB individuals. Conceptual frameworks including minority stress theory and social safety theory support the hypothesis that the stress of experienced racial discrimination produces social threat, which in turn perpetuates racial disparities in chronic pain (i.e., physical pain).. These findings provide implications for potential therapeutic targets for the management and treatment of chronic pain conditions.

## Declaration of Competing Interest

The authors declare that they have no known competing financial interests or personal relationships that could have appeared to influence the work reported in this paper.
